# Management of Advanced Tricompartmental Knee Osteoarthritis With Degenerative Meniscal Pathology Using Extracorporeal Magnetotransduction Therapy, Extracorporeal Shockwave Therapy, Microfragmented Adipose Tissue, and Platelet-Rich Plasma

**DOI:** 10.7759/cureus.116369

**Published:** 2026-09-16

**Authors:** Aneesh Garg, Spencer Decker, Micky Nguyen

**Affiliations:** 1 Sports Medicine and Regenerative Orthopaedics, Rocky Vista University College of Osteopathic Medicine, Boulder, USA; 2 Regenerative Medicine, Dynamic Athlete, Boulder, USA

**Keywords:** degenerative meniscus, emtt, eswt, joint preservation, knee osteoarthritis, mfat, microfragmented adipose tissue, non-surgical interventions, prp, shockwave

## Abstract

Advanced tricompartmental knee osteoarthritis (OA) with degenerative meniscal tears and ligamentous insufficiency represents a considerably difficult presentation in non-surgical musculoskeletal medicine. Although total knee arthroplasty is the current standard for surgical intervention, many patients may prefer to first explore non-surgical options or may require interim symptom management prior to surgery. This report describes management of a complex case of advanced knee OA via a staged non-surgical protocol utilizing extracorporeal shockwave therapy (ESWT), extracorporeal magnetotransduction therapy (EMTT), and a combined microfragmented adipose tissue (MFAT) and platelet-rich plasma (PRP) procedure in a 68-year-old female. The Knee Injury and Osteoarthritis Outcome Score (KOOS) and Numeric Rating Scale (NRS) were used to track patient progress and pain throughout treatment and post-procedure follow-ups. The KOOS is a patient-reported outcome measure consisting of five subscales in its entirety: pain, symptoms, activities of daily living, recreation function, and quality of life (QOL). For the purposes of tracking our outcomes in this specific case, we determined that only the pain, symptoms, and QOL subscales were relevant to the real-world outcomes of this patient. The NRS is an 11-point patient-reported pain scale ranging from 0 to 10. The patient showed a reduction in NRS pain scores and overall improvement in KOOS domains of pain, symptoms, and QOL. Biologic joint preservation using MFAT and PRP with mechanotransductive priming produced clinically meaningful improvement in advanced knee OA, warranting consideration as a bridge or alternative to arthroplasty in appropriate patients.

## Introduction

Tricompartmental knee osteoarthritis (OA) is a condition wherein the degenerative process has reached all three compartments of the knee and affects up to 17% of patients presenting for surgical evaluation [1]. When combined with meniscal tears and anterior cruciate ligament (ACL) deficiency, as in this case, it creates a complex mechanical and biological problem that cannot be resolved with a single injection or course of physical therapy alone. ACL insufficiency in particular produces progressive intra-articular damage through altered joint mechanics, accelerating cartilage loss and meniscal degeneration over time [2]. Although total knee arthroplasty works well for many cases like this, many patients are not prepared for that step. Current non-surgical management methods for managing this pathology, including physical therapy, corticosteroids, and over-the-counter medications, primarily aim to reduce symptoms in an effort to increase function. However, these interventions do not address the progressive cartilage loss or meniscal degeneration.

Microfragmented adipose tissue (MFAT) is harvested through a minimally invasive aspiration procedure and processed mechanically without enzymes, preserving the stromal vascular niche and protecting the mesenchymal stem cells within it [3]. This delivers adipose-derived stromal cells, growth factors, and extracellular matrix components directly to the joint. When combined with platelet-rich plasma (PRP) and preceded by mechanotransductive priming with extracorporeal magnetotransductive therapy (EMTT) and extracorporeal shockwave therapy (ESWT), the protocol addresses multiple aspects of the problem simultaneously. ESWT acts through mechanotransduction to produce biological responses in target tissues, including anti-inflammatory effects, promotion of cell proliferation, and stimulation of neovascularization, which may help prime the joint environment prior to biologic injection [4].

## Case presentation

A 68-year-old female with a body mass index (BMI) of 31.1 kg/m^2^ presented with a history of inflammatory arthritis, OA, and osteoporosis, and chronic bilateral medial and lateral knee pain that was worse on the left and on the medial aspect. No pertinent surgical or injection history of the bilateral knees. She traced the onset back to a significant ski injury from early adulthood, with pain progressively worsening over the years. She described a mix of aching, dull, and sharp pain that flared with walking, standing, climbing stairs, and twisting movements. Morning stiffness was present but tended to improve as the day went on. Prior conservative management included heat, massage therapy, formal physical therapy, non-steroidal anti-inflammatory drugs, and Tylenol. The patient reported completing more than six consecutive weeks of physical therapy multiple times from 2022 to 2025 with limited to no improvements in symptoms. No pain medications were prescribed. The patient reports taking Tylenol when pain flares were severe. For patients with significant comorbidities and those who have exhausted conservative options without adequate relief, targeted biologic and physical therapies represent an important emerging treatment category [5].

X-ray imaging and MRI of the bilateral knees showed advanced tricompartmental OA on the left, classified as Kellgren-Lawrence grade 4, with the worst changes in the medial compartment, along with degenerative medial and lateral meniscal tears and chronic ACL deficiency of the left knee. The right knee demonstrated Kellgren-Lawrence grade 3 OA (Figures 1-2).

**Figure 1:**
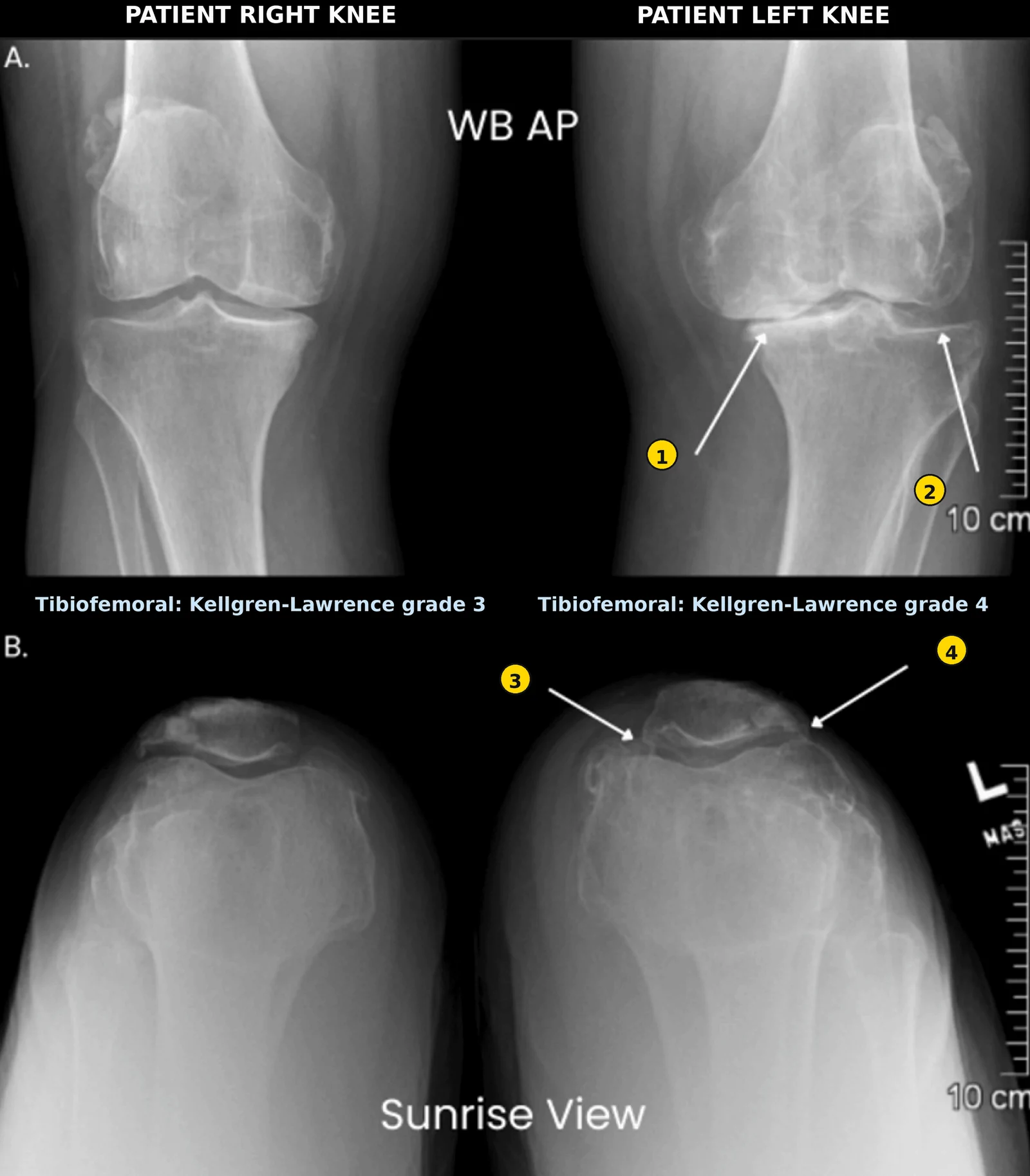
AP Weightbearing and Sunrise View Bilateral Knee X-rays WB = weightbearing, AP = anteroposterior Patient's right knee is shown on the viewer's left in both panels. (A) Weightbearing AP view. The left knee demonstrates Kellgren-Lawrence grade 4 tibiofemoral osteoarthritis; the right knee demonstrates grade 3. Arrow 1 indicates medial compartment joint space narrowing with subchondral sclerosis. Arrow 2 indicates lateral compartment joint space narrowing. (B) Sunrise view demonstrating patellofemoral compartment osteoarthritis bilaterally, more severe in the left knee. Arrows 3 and 4 indicate patellofemoral joint space narrowing with marginal osteophyte formation.

**Figure 2:**
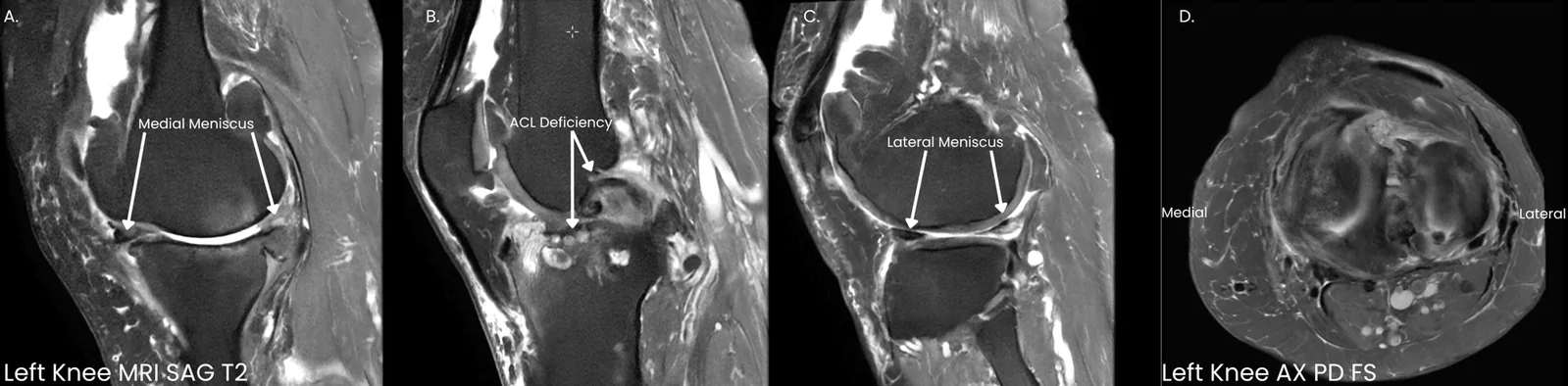
MRI Without Contrast of the Left Knee Sagittal and Axial Views SAG = sagittal, T2 = T2-weighted image, ACL = anterior cruciate ligament, AX = axial, PD = proton density, FS = fat saturation, MRI = magnetic resonance imaging (A) Medial view showing severe medial compartment osteoarthritis with tearing to the medial meniscus. (B) Intermediate view showing chronic full-thickness anterior cruciate ligament tear. (C) Lateral View showing moderate lateral compartment osteoarthritis and complex tearing of the lateral meniscal posterior horn. (D) Axial view showing complex tearing and maceration of the medial meniscus. Complex tearing of the lateral meniscal posterior horn. Small intra-articular body in the lateral joint compartment.

On examination, tenderness along the medial and lateral joint lines of the left knee was appreciated, mild effusion, reduced flexion and extension of the knee with active and passive range of motion, and mild varus alignment bilaterally.

The treatment plan devised consisted of three phases: mechanotransductive priming, biologic intervention, and rehabilitation. Based on the patient's history, clinical presentation, resulting imaging, and physical examination, a treatment plan was created to target the left knee, as this joint was more symptomatic than the right. The ESWT and EMTT course consisted of six total visits. Each visit started with EMTT (MAGNETOLITH®, STORZ MEDICAL AG, Tägerwilen, Switzerland) to the medial joint line of the left knee, followed by ESWT (DUOLITH® SD1 T-TOP, STORZ MEDICAL AG, Tägerwilen, Switzerland) to the same region. The decision to target the medial joint line was based on the severity of the OA in the medial compartment as seen on X-ray imaging, and supported by evidence demonstrating that ESWT applied to the medial tibial subchondral bone produces significantly greater chondroprotective effects than application to lateral or femoral locations, reducing gross pathological OA changes, cartilage defect area, and improving subchondral bone microarchitecture [6]. The energy levels of the ESWT were titrated up each visit based on patient tolerance. Both the Standoff 1 (S1) and Standoff 2 (S2) were used to achieve deeper or more superficial penetration, respectively, as needed. With both standoffs, the energy levels started at 0.05 mJ/mm^2^ and were titrated up to 0.25 mJ/mm^2^ by the end of the treatment course. These sessions were aimed at reducing pain, improving the joint-level tissue environment, and beginning to address mechanical irritation. Throughout the mechanotransductive priming phase, a home-based hip and core strengthening program was followed, and activity was modified to avoid aggravating the knee further.

Following the mechanotransductive priming phase, the patient underwent biologic intervention consisting of a same-day MFAT and PRP procedure. Using a Cervos KeyPRP™ kit (Cervos Medical LLC, Avon, MA), the platelet injections were prepared from her own blood taken from the anterior aspect of her elbow. The MFAT was harvested from the bilateral flanks using a Cervos LIPO-PRO™ adipose processing system. Prior to the injections, the joint was visualized under ultrasound (US) imaging. Ultrasound-guided intra-articular injection has been shown to be significantly more accurate than landmark-guided injection, and importantly, ultrasound guidance increases effusion detection and the volume of fluid successfully aspirated, making it particularly valuable when pre-injection aspiration is warranted [7]. An effusion of the left knee was appreciated on the US and aspirated before introducing the orthobiologics, which yielded 15.0 cc of clear, straw-colored fluid. The aspirated fluid was not submitted for laboratory or cytologic analysis. Both orthobiologic products were prepared according to the manufacturer's validated closed-system protocol; no independent platelet count, leukocyte count, or cell viability assessment was performed on either preparation. Both products were delivered intra-articularly to the left knee under US guidance, consistent with evidence supporting ultrasound-guided delivery as the preferred approach for accurate and reproducible intra-articular access [8]. Strict sterile protocols were followed throughout the entirety of the procedure.

Postoperatively, the patient was instructed to only bear roughly 25% of her weight on the left lower extremity for 72 hours. A walker was provided in the place of crutches due to the patient's instability on crutches. Formal physical therapy was to restart three weeks status post injection; however, the patient was unable to be seen by a physical therapist until two months post procedure. The patient was instructed to continue with prior at-home exercise programs to fill the gap before formal physical therapy sessions started.

Throughout the course of treatment and during follow-up visits, the patient's progress was tracked using NRS rating and KOOS subscales [9,10]. The NRS is an 11-point segmented numeric scale ranging from 0, which represents no pain, to 10, representing the worst pain imaginable. The patient selects the whole number that best reflects their current pain intensity. This format has been demonstrated to perform better than both simple descriptive scales and continuous visual analog scales for clinical pain measurement [9]. The KOOS is a self-administered, patient-reported outcome measure developed to assess short- to long-term outcomes following knee injury and knee OA across five subscales: pain, symptoms, activities of daily living, sport and recreation function, and knee-related quality of life (QOL). Each subscale is scored from 0 to 100, where higher scores reflect fewer symptoms and better function [10]. The KOOS has demonstrated reliability, validity, and responsiveness to both surgical and non-surgical interventions and has been specifically validated in populations with ACL injury, meniscal pathology, and OA [10]. When implementing the KOOS subscales into the clinical workflow, it was determined to only administer the symptoms, pain, and QOL subscales to the patient. This does not affect the scoring of the KOOS as each subscale is scored individually and can be presented as such. At initial consult, baseline KOOS subscale scores were recorded with symptoms of 32.1, pain of 36.1, and QOL of 6.3, and an NRS of 4/10. Scores were tracked at each treatment visit and follow-up appointment, as detailed in Table 1, Figure 3, and Figure 4. 

**Table 1:** Treatment and Follow-Up Timeline Subscale Scores QOL = quality of life, NRS = numerical rating score, Δ = absolute change from baseline in KOOS points Study day is counted from the initial consultation. "d post" is elapsed days from the MFAT/PRP procedure on study day 54. KOOS subscales scored 0 to 100; higher scores indicate fewer symptoms and better function. NRS scored 0 to 10; lower is better. n/s = not scheduled per standard outcome-collection protocol, under which the questionnaire is administered at the consultation and first treatment, at the third and sixth treatments, and at all post-procedure follow-ups; NRS is recorded at every visit.

Timepoint	Timing	Symptoms	Δ	Pain	Δ	QOL	Δ	NRS
Consultation, baseline	Day 0	32.14	—	36.11	—	6.25	—	4
Treatment 1	Day 34	32.14	0	27.78	-8.33	0	-6.25	6
Treatment 2	Day 39	n/s	n/s	n/s	n/s	n/s	n/s	4
Treatment 3	Day 42	42.86	10.72	22.22	-13.89	12.5	6.25	4
Treatment 4	Day 46	n/s	n/s	n/s	n/s	n/s	n/s	4
Treatment 5	Day 49	n/s	n/s	n/s	n/s	n/s	n/s	4
Treatment 6	Day 52	28.57	-3.57	27.78	-8.33	18.75	12.5	4
MFAT/PRP procedure	Day 54	—	—	—	—	—	—	4
Follow-up 1	Day 66, 12 d post	n/s	n/s	n/s	n/s	n/s	n/s	6
Follow-up 2	Day 81, 27 d post	64.29	32.15	55.56	19.45	37.5	31.25	2
Follow-up 3	Day 101, 47 d post	71.43	39.29	61.11	25	62.5	56.25	0
Follow-up 4	Day 220, 166 d post	60.71	28.57	58.33	22.22	62.5	56.25	0
Follow-up 5	Day 297, 243 d post	67.86	35.72	69.44	33.33	62.5	56.25	0

**Figure 3:**
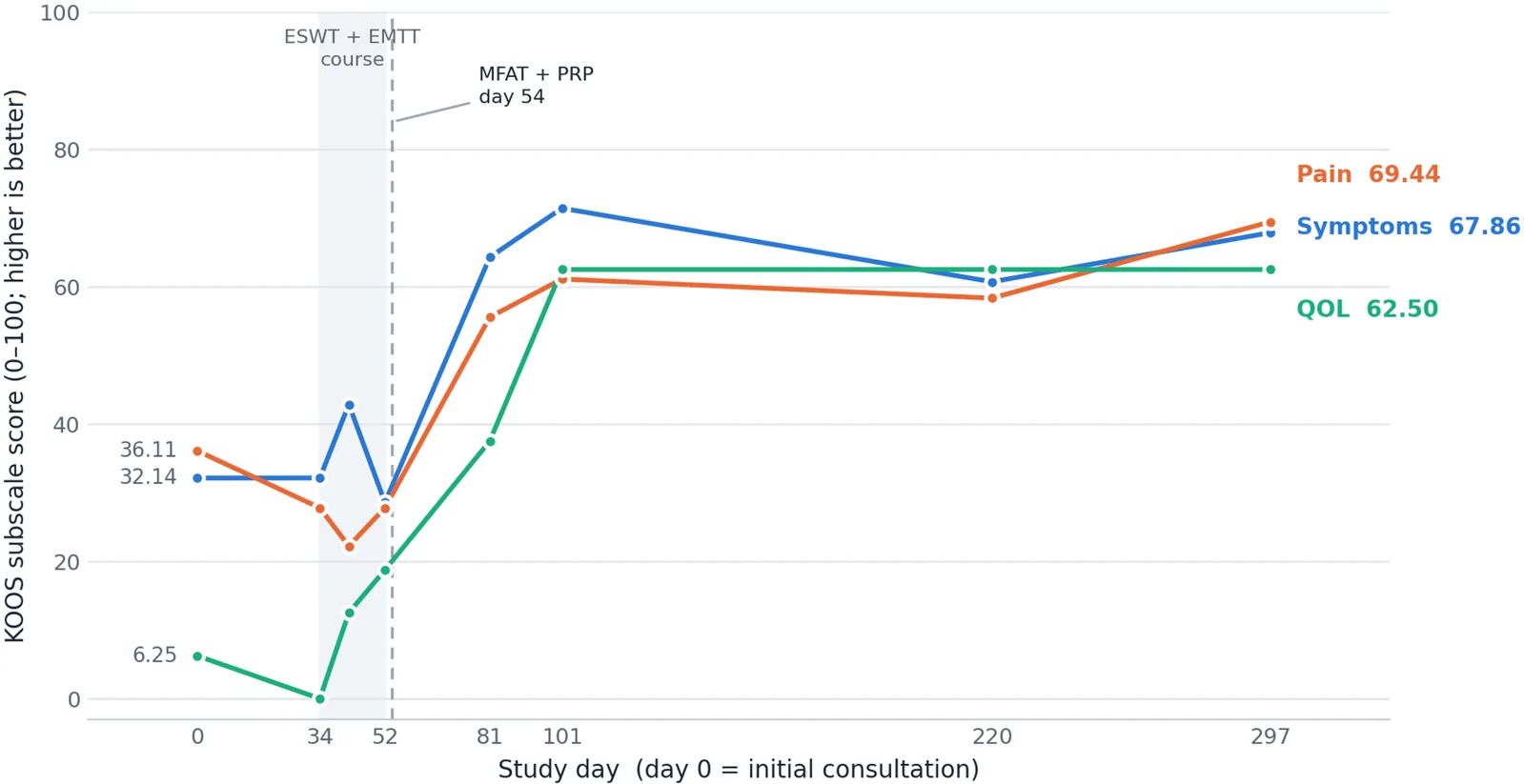
KOOS Symptoms, Pain, and QOL Subscale Scores Over Time Subscales are scored 0-100, with higher scores indicating fewer symptoms and better function. The shaded span marks the course of extracorporeal shockwave and magnetotransduction therapy from study day 34 to study day 52, and the dashed line marks the microfragmented adipose tissue and platelet-rich plasma injection on study Day 54. The horizontal axis is scaled to elapsed study day. Subscale scores were not obtained at treatments 2, 4, and 5 or at the 12-day post-injection visit, and the plotted lines interpolate across those intervals. Baseline scores were 32.14 for symptoms, 36.11 for pain, and 6.25 for quality of life. Scores were unchanged or below baseline through the mechanotransductive course, with pain reaching a nadir of 22.22 at study Day 42, and rose across all three subscales following the injection. A partial decline in symptoms and pain was recorded at study Day 220 before both recovered by study Day 297, when final scores were 67.86 for symptoms, 69.44 for pain, and 62.50 for quality of life. ESWT = extracorporeal shockwave therapy, EMTT = extracorporeal magnetotransduction therapy; KOOS = Knee Injury and Osteoarthritis Outcome Score; MFAT = microfragmented adipose tissue; QOL = quality of life

**Figure 4:**
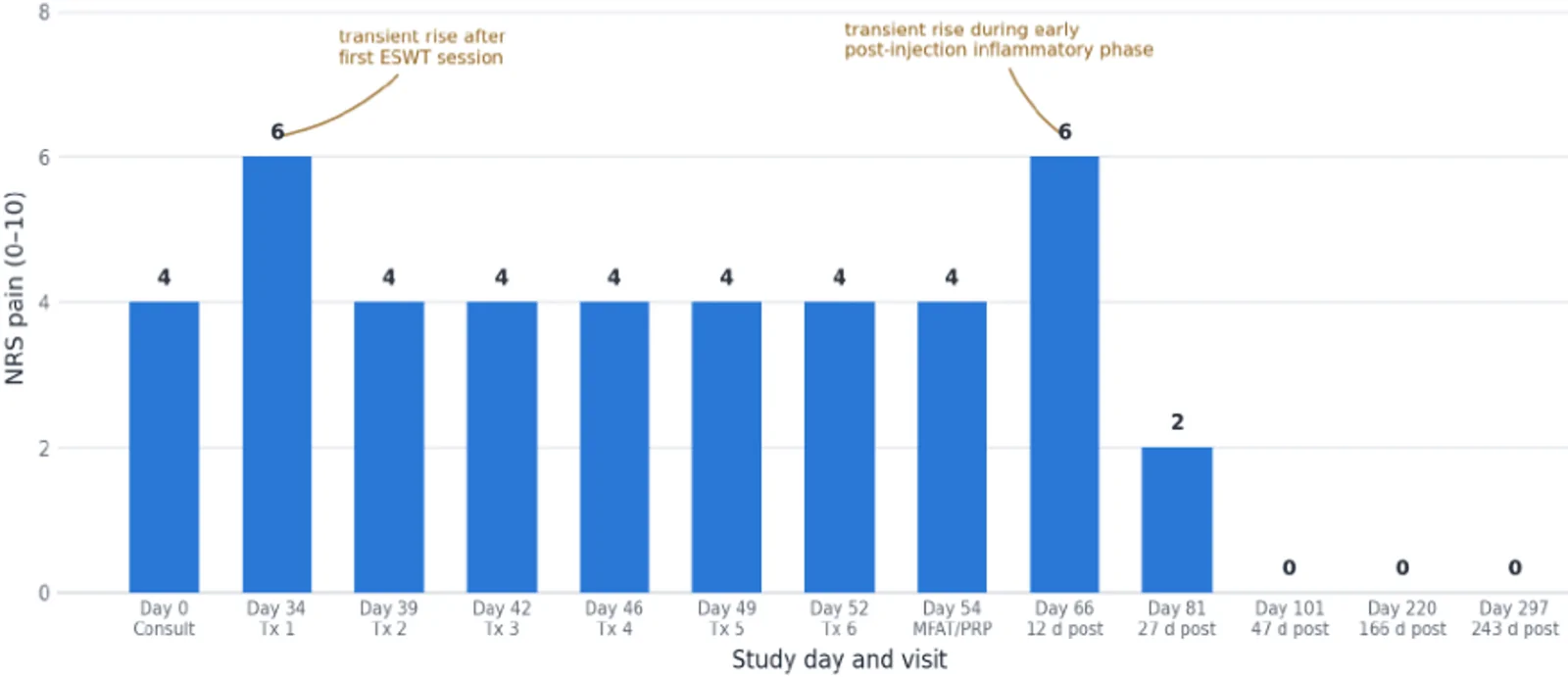
Change in the NRS Over Time Numeric Rating Scale (NRS) pain scores from the initial consultation through the final follow-up at study Day 297. NRS is scored 0 to 10, with higher scores indicating greater pain. Unlike the KOOS subscales, NRS was recorded at every visit. Pain was 4 at baseline, rose transiently to 6 following the first extracorporeal shockwave and magnetotransduction session, and returned to 4 for the remainder of the mechanotransductive course. The score recorded on the day of the microfragmented adipose tissue and platelet-rich plasma injection reflects status on presentation, prior to the procedure. A second transient rise to 6 was recorded 12 days after injection during the early post-injection inflammatory phase, followed by a decline to 2 at 27 days and to 0 at 47 days, which was sustained at 166 and 243 days. ESWT = extracorporeal shockwave therapy; KOOS = Knee Injury and Osteoarthritis Outcome Score

## Discussion

Early traumatic knee injuries, particularly those involving the ACL and meniscus, are well-established potent risk factors for the development of post-traumatic OA (PTOA), with reported incidence as high as 87% following ACL injury in cases involving concomitant meniscal damage. Particularly for patients who have exhausted conservative options without adequate relief or those with significant comorbidities, targeted biologic and physical therapies represent an important emerging treatment category to modulate the intra-articular environment involved in this complex pathology.

Several features of this protocol warrant particular attention. The use of mechanotransductive priming with EMTT and ESWT prior to orthobiologic injection represents a deliberate effort to optimize the joint environment before introducing regenerative agents. EMTT utilizes a rapidly changing electromagnetic field that induces electrical currents within tissue, which may modulate cell membrane potential and ion channel activity and thereby influence cellular signaling. In contrast, ESWT emits focused acoustic waves that carry energy into the targeted tissue. This mechanical stimulus activates mechanotransduction pathways, which can influence biochemical signaling and promote therapeutic effects, such as analgesia, cellular proliferation, and neovascularization. By targeting the medial tibial subchondral bone with ESWT, the site shown to produce the greatest chondroprotective response in OA knees, the priming phase aimed to reduce local inflammation, stimulate neovascularization, and enhance tissue receptivity prior to MFAT and PRP injection. Ultrasound guidance throughout the injection procedure, including pre-injection aspiration of 15 cc of effusion, ensured accurate intra-articular delivery of both products and allowed real-time assessment of the joint environment prior to orthobiologic administration. The combination of MFAT and PRP delivered together in a single same-day procedure provided a complementary biological payload. MFAT possesses potent anti-inflammatory properties and contains the highest concentrations of mesenchymal stem cells among tissues in the body. MFAT also introduces growth factors and extracellular matrix components, whereas PRP provides a concentrated autologous source of platelet-derived growth factors. These injectables, used concurrently, modulate the local inflammatory and anabolic environment, inducing beneficial cellular processes.

At initial consult, the patient demonstrated severe symptom burden and functional impairment, with KOOS symptoms, pain, and QOL subscale scores of 32.1, 36.1, and 6.3, respectively (see Table 1, Figure 3), accompanied by an NRS pain score of 4/10. These values underscore the substantial impact that this chronic knee pathology had on the patient's daily life and functional confidence despite multiple attempts at conservative management. To contextualize the clinical significance of changes observed throughout this case, previously established minimal clinically important difference (MCID) thresholds for KOOS subscales in knee OA injection populations are approximately 8-10 points for pain and QOL, with over 85% of patients achieving MCID at both 6 and 12 months post injection in biologic treatment cohorts [11].

During the mechanotransductive priming phase preceding orthobiologic injection, the patient showed early but modest changes prior to orthobiologic treatment. KOOS symptoms ranged from 32.1 to 42.9 and pain ranged from 22.2 to 27.8 across treatment visits, changes consistent with gradual neuromodulation and improved load tolerance in response to the EMTT and ESWT protocol. QOL remained low during this phase, ranging from 0.0 to 18.8, which is not unexpected given the short duration and the preparatory rather than regenerative nature of this phase. NRS stabilized at 4/10 from Treatment 2 onward through the MFAT procedure day, suggesting that the patient reached a pain floor during the active treatment phase that neither worsened nor improved acutely. Treatment 1 produced a transient NRS spike to 6/10. However, this is consistent with the expected initial tissue response to ESWT. Shockwave therapy may produce a temporary increase in local discomfort during the first one to two treatment sessions as a result of the mechanobiological activation process, mediated in part by an initial increase in substance P release at the treatment site, and should therefore not be mistakenly interpreted as a negative treatment response [12,13].

A second transient increase in discomfort occurred during the early post-injection period. Upon 10-day post-injection follow-up evaluation, NRS increased to 6/10. This is a clinically coherent finding consistent with the known inflammatory response following intra-articular biologic injection. Pain, swelling, and stiffness have been reported in up to 79% of treated knees after MFAT administration, typically resolving spontaneously within approximately 16.6 days and without impairing longer-term clinical outcomes [14]. Consequently, this early post-procedural reaction may therefore be contextualized as part of the expected biological cascade rather than treatment failure.

Once this initial inflammatory period resolved, a substantial clinical shift occurred. By the three-week post-injection follow-up, KOOS symptoms had improved to 64.3, pain to 55.6, and QOL to 37.5, representing gains of 100%, 53.9%, and 500% over baseline, respectively. Each of these improvements substantially exceeds published MCID thresholds, indicating meaningful functional gains following the combined mechanotransductive and biologic intervention [11]. NRS pain had decreased substantially to 2/10 at this visit, reflecting a rapid and progressive recovery from the early post-procedural response and the beginning of a sustained upward trajectory. This pattern of rapid improvement following the post-injection inflammatory window aligns with the biological mechanism of MFAT, wherein adipose-derived mesenchymal stem cells exert paracrine anti-inflammatory and tissue-regenerative effects that become clinically apparent once the initial acute response resolves [3].

The six-week follow-up revealed that the patient had achieved her highest functional status to that point, with KOOS symptoms of 71.4, pain of 61.1, and QOL of 62.5, representing improvements of 122.2%, 69.2%, and 900% over baseline values, respectively; each reflecting clinically significant change across all measured domains and far exceeding established MCID thresholds [11]. NRS reached 0/10 at this visit, representing a complete absence of in-clinic pain at the time of assessment.

Although KOOS scores at the six-month follow-up demonstrated a modest decline from their six-week peak, the patient maintained substantial improvement across every measured domain. KOOS symptoms of 60.7, pain of 58.3, and QOL of 62.5, representing improvements of 88.9%, 61.5%, and 900% over baseline, respectively. NRS remained at 0/10. This slight plateau is consistent with the natural trajectory observed in biologic interventions, in which the period of active tissue remodeling gives way to a more stable phase. This is a pattern that has been documented in real-world MFAT data, where NRS demonstrates a prompt reduction at six months, followed by modest fluctuation at subsequent time points while remaining well below baseline [13,15]. These results indicate that, despite the modest KOOS fluctuation, the patient's pain burden had been eliminated and her global sense of functional well-being had been nearly fully restored. By the eight-month follow-up, the patient demonstrated continued improvement across all KOOS subscales, with symptoms reaching 67.9, pain reaching 69.4, and QOL remaining at 62.5. The KOOS pain subscale improvement of 92.3% over baseline is particularly notable, as pain reduction is often the primary driver of functional recovery and patient-reported satisfaction in this population. NRS remained at 0/10, sustained now across two consecutive follow-up time points spanning a six-month window, which demonstrates durable symptom relief.

Throughout the treatment timeline, the NRS, percent feeling overall, and KOOS subscale data tell a coherent and compelling clinical story. A predictable and transient early pain response was observed during both the priming and immediate post-injection phases, each consistent with well-characterized biological responses to ESWT and intra-articular biologic delivery, respectively [12,14], followed by progressive and durable improvement that culminated in a near-complete resolution of pain by six weeks, which was sustained through eight months. The 0/10 NRS at both the six- and eight-month time points, combined with KOOS improvements that consistently and substantially exceeded MCID thresholds [11], reflects a meaningful and durable therapeutic response to the combined EMTT/ESWT priming and same-day MFAT/PRP intervention strategy in a patient who entered treatment at 30% global well-being after failing multiple prior conservative interventions. These findings are consistent with the broader MFAT literature showing that 81% of patients experience clinically meaningful pain reduction and functional improvement following a single intra-articular MFAT injection [3].

This case report should be interpreted with explicit limitations. As a single-patient case report without a control arm, causality cannot be established between treatment methods and patient-observed improvements. Additionally, the relatively short observation period of eight months limits the generalizability of long-term durability of outcomes. It is important to mention that the patient did not begin formal physical therapy until approximately two months following the procedure; the intended start date was three weeks post procedure. This delay may have influenced the pace and magnitude of improvement, among other factors. Although patient-reported outcome measures demonstrated substantial improvement across measured domains, advanced imaging was not obtained after treatment to determine physiological structural change. Due to the combined multimodal treatment approach, it is difficult to determine the independent contribution of each treatment method. Nevertheless, the magnitude and persistence of improvement observed in this patient provide valuable insight into the potential therapeutic role of this combined intervention strategy and support the need for further investigation in larger, controlled studies.

Other nonsurgical approaches have been reported for advanced knee OA, including culture-expanded and bone marrow-derived cell preparations, prolotherapy and dextrose-based injection, and nutritional and metabolic optimization. The comparative evidence base does not currently support ranking these approaches against one another, and a single case cannot contribute to that comparison. The protocol reported here is therefore presented as one described approach rather than as a preferred one.

## Conclusions

This case report should be interpreted with explicit limitations. As a single patient receiving a multimodal protocol without a control arm, causality cannot be established between any treatment component and the observed improvement, and no individual component can be isolated. The observed change may reflect any combination of the individual modalities, natural fluctuation of symptoms, contextual and placebo effects, mechanical decompression from aspiration of 15.0 cc of effusion, activity modification and unloading, the home exercise program, the timing of formal rehabilitation, or regression to the mean. The patient's inflammatory arthritis history warrants particular emphasis, as fluctuation in inflammatory disease activity is an alternative explanation for the observed trajectory.

Several elements of the protocol are incompletely characterized. Pulse count and pulse frequency were not documented on a per-session basis in the clinical record and are therefore not reported; energy was titrated to patient tolerance rather than fixed in advance, so no predetermined parameter set existed for this course. Platelet concentration, leukocyte content, and cell viability were not measured for either orthobiologic preparation, which limits reproducibility. The aspirated synovial fluid was not submitted for analysis. Nutritional and metabolic laboratory values were not obtained as part of this patient's care. Outcome measurement was also partial. Only the symptoms, pain, and QOL subscales of the KOOS were administered. Each was administered in full, comprising all seven symptom items, all nine pain items, and all four QOL items, so the reported scores are complete and scored as the instrument intends. However, the activities of daily living and sport and recreation subscales were not administered, so no total or composite KOOS value is calculable, and function was not captured by any patient-reported measure in this case. The baseline questionnaire was completed at the initial consultation, 34 days before the first mechanotransductive session and 54 days before the injection. The MCID and patient acceptable symptom state (PASS) values used for interpretation were derived from intra-articular injection cohorts and are not validated for this protocol. Finally, the observation period of approximately eight months limits conclusions about long-term durability, and no post-treatment imaging was obtained, so no structural change can be demonstrated. Further investigation in larger controlled studies is required before any inference about efficacy can be drawn.
